# Supplementary material for: Creation of a non-Western humanized gnotobiotic mouse model through the transplantation of rural African fecal microbiota
Source: Microbiol Spectr. 2023 Oct 11;11(6):e01554-23. doi: 10.1128/spectrum.01554-23 (PMC10714993; doi:10.1128/spectrum.01554-23)
Supplement: Supplemental figures and tables — Fig. S1 to S7 and Tables S1 and S4 to S6. [file spectrum.01554-23-s0001.pdf]

Creation of a non-Western humanized gnotobiotic mouse model through the transplantation of rural African fecal microbiota

Kristin M. Van Den Ham[1], Morgan R. Little[1], Olivia J. Bednarski[1], Elizabeth M. Fusco[1], Rabindra K. Mandal[1], Riten Mitra[2], Shanping Li[3], Safiatou Doumbo[4], Didier Doumtabe[4], Kassoum Kayentao[4], Aissata Ongoiba[4], Boubacar Traore[4], Peter D. Crompton[3], Nathan W. Schmidt[1]*

[1]Ryan White Center for Pediatric Infectious Diseases and Global Health, Herman B. Wells Center for Pediatric Research, Department of Pediatrics, Indiana University School of Medicine, Indianapolis, Indiana, United States of America
[2]Department of Bioinformatics and Biostatistics, University of Louisville, Louisville, Kentucky, United States of America
[3]Malaria Infection Biology and Immunity Section, Laboratory of Immunogenetics, National Institute of Allergy and Infectious Diseases, National Institutes of Health, Rockville, Maryland, United States of America
[4]Mali International Center of Excellence in Research; Malaria Research and Training Center, University of Sciences, Techniques and Technologies of Bamako, Bamako, Mali
*Corresponding author
E-mail: nwschmid@iu.edu (NWS)

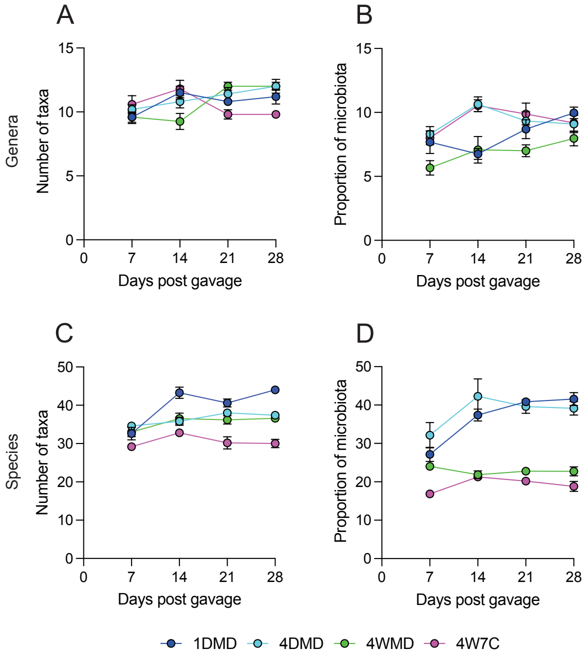

Figure S1. Genus- and species-level taxa detected in the mice but not in the input
The number of genus- (A) and species-level taxa (C) and the proportion of the microbiome accounted for by those genus- (B) and species-level taxa (D) detected in the murine samples but not in the input in each mouse group over time. Mean values ± SEM are plotted.

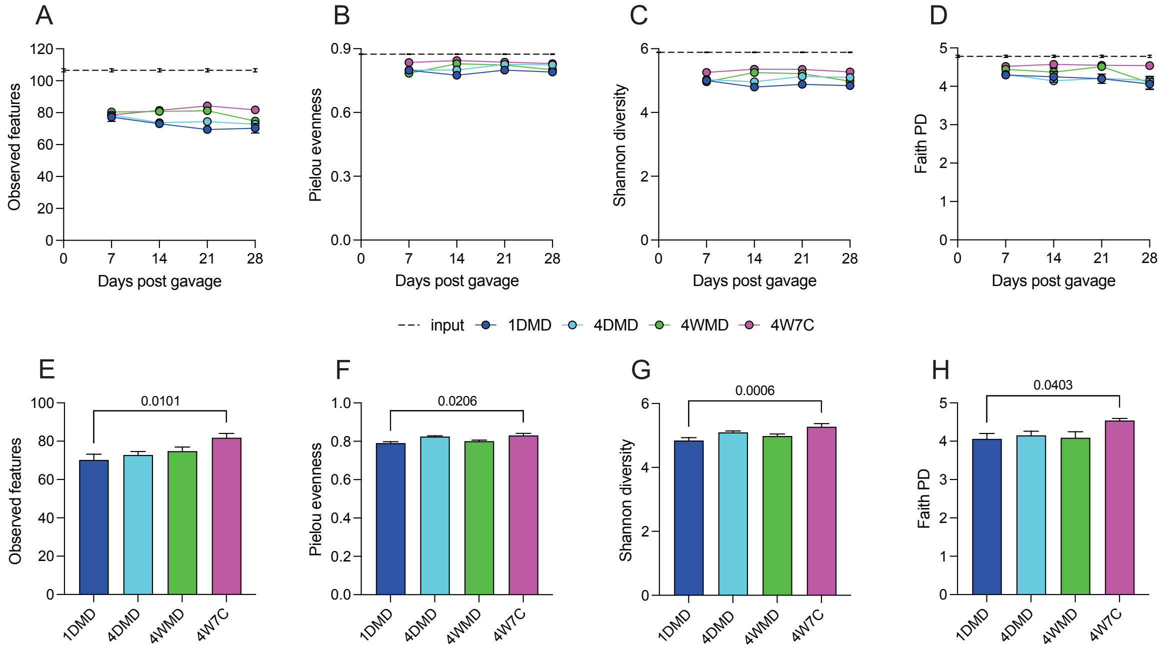

Figure S2. Alpha diversity of the gut microbiome

The number of observed features (A), Pielou evenness (B), Shannon diversity (C) and Faith PD (D) of the input and the engraftment groups. Observed features (E), Pielou evenness (F), Shannon diversity (G), and Faith PD (H) of the engraftment groups on d28 was compared using ANOVA with Tukey's multiple comparison test. Mean values ± SEM are plotted.

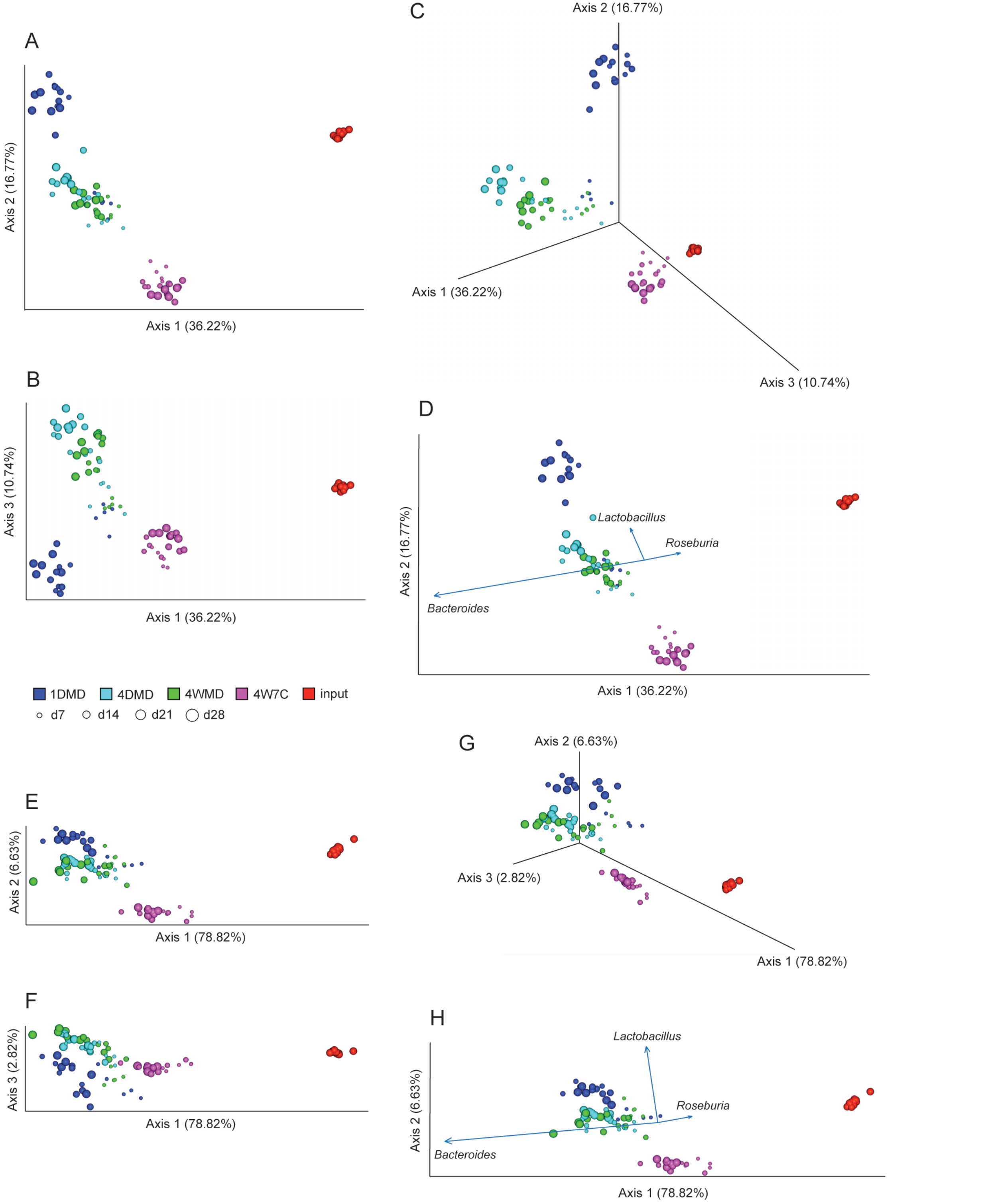

Figure S3. Jaccard and weighted Unifrac diversity

PCoA plots based on the Jaccard distance (A-C) and weighted Unifrac distance (E-G). Biplot of the top three genera driving ordination using Jaccard distance (D) and weighted Unifrac distance (H).

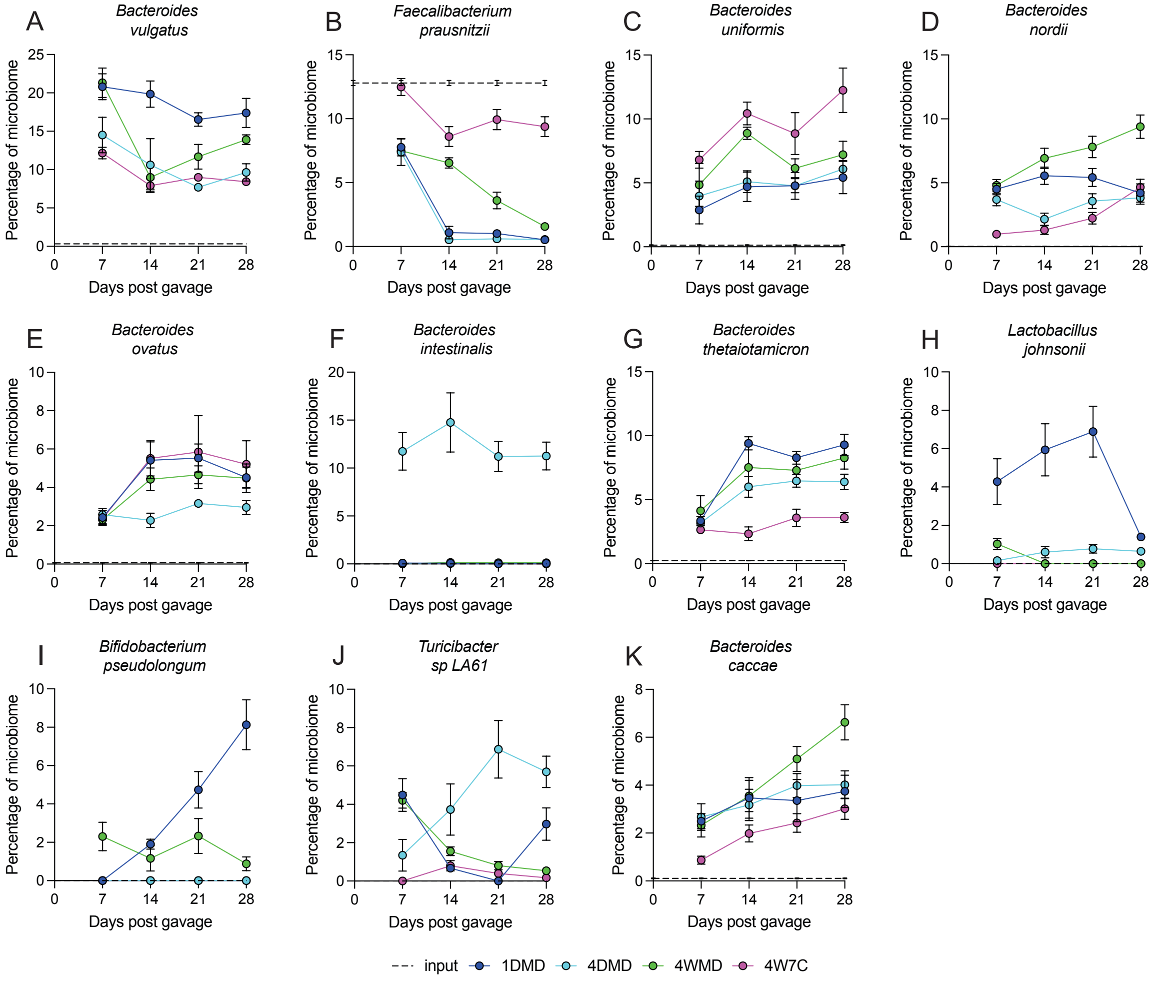

Figure S4. Principal species driving beta-diversity ordination

The top ten species driving ordination were determined using Jaccard distance, Bray-Curtis distance, unweighted Unifrac distance, and weighted Unifrac distance. *B. vulgatus*, *F. prausnitzii*, *B. uniformis*, *B. nordii*, *B. ovatus* and *B. intestinalis* were major drivers for all four metrics (A-F). *B. thetaiotaomicron* and *L. johnsonii* were major drivers for Jaccard distance, unweighted Unifrac distance and weighted Unifrac distance (G and H). *Bifidobacterium pseudolongum* was a major driver for Jaccard distance, Bray-Curtis distance, and unweighted Unifrac distance (I). *Turicibacter* sp LA61 was a major driver for Bray-Curtis distance, unweighted Unifrac distance and weighted Unifrac distance (J). *B. caccae* was a major driver for Bray-Curtis distance and weighted Unifrac distance (K). *Lactobacillus gasseri* was only a major driver using Bray-Curtis distance and *Dorea longicatena* was only a major driver using Jaccard distance and are not shown. Mean values ± SEM are plotted.

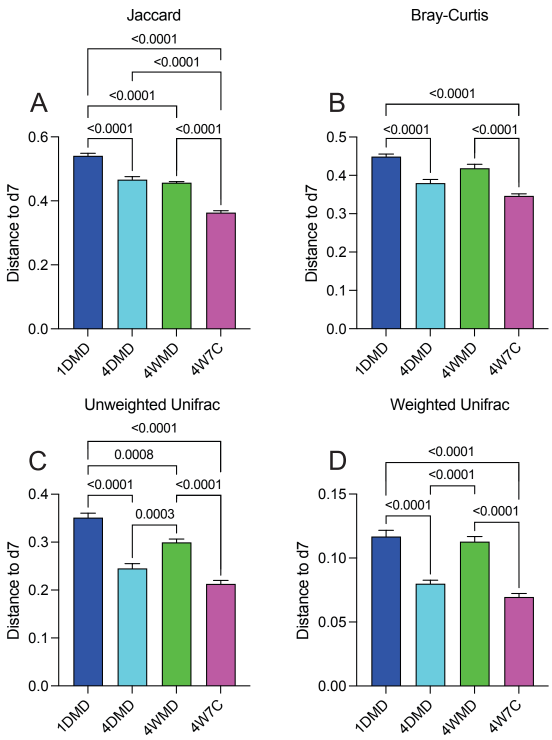

Figure S5. Distance between day 7 and day 28

The distance between d7 and d28 for each group using Jaccard distance (A), Bray-Curtis distance (B), unweighted Unifrac distance (C), and the weighted Unifrac distance (D) was compared using ANOVA with Tukey's multiple comparison test. Mean values ± SEM are plotted.

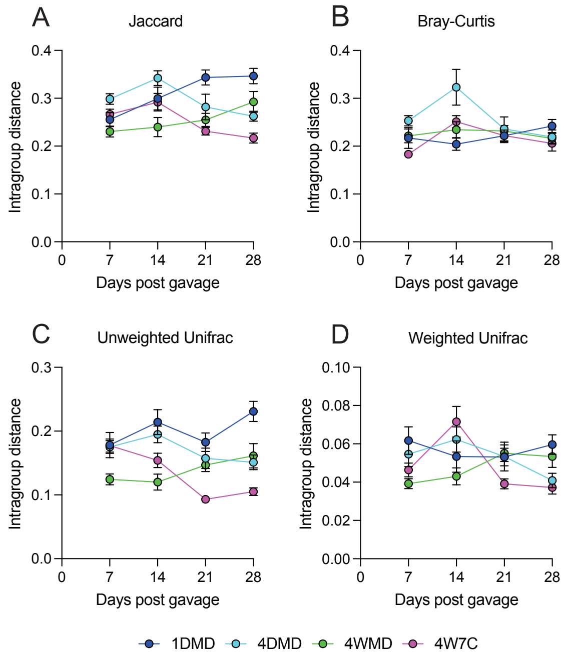

Figure S6. Longitudinal intragroup variation of the microbiome

The intragroup distance at each time point for each gavage group, using Jaccard distance (A), Bray-Curtis distance (B), unweighted Unifrac distance (C) and the weighted Unifrac distance (D). Mean values ± SEM are plotted.

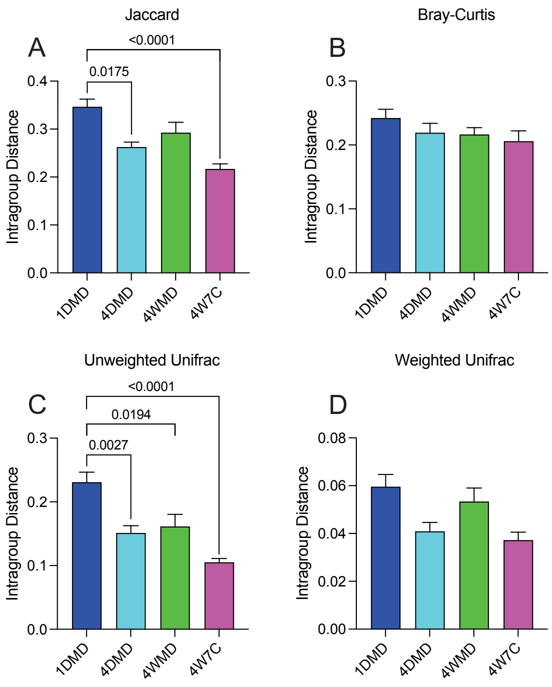

Figure S7. Intragroup variation on day 28

The intragroup distance for each group at d28 using Jaccard distance (A), Bray-Curtis distance (B), unweighted Unifrac distance (C) and the weighted Unifrac distance (D), was compared using ANOVA with Tukey's multiple comparison test. Mean values ± SEM are plotted.

Table S1A. Ingredient composition of Malian diet

Ingredient	Percent of diet (w/w)	Amount in 5000g (g)
millet	28.31	1415
sorghum	28.31	1415
kefir	10.28	514
tomatoes	6.03	302
sweet potato	4.78	239
cabbage	3.77	188
red bell pepper	3.77	188
kale:chard:collard greens:turnip greens:parsley (1:1:1:1:1)	3.68	184
kidney beans	3.35	168
great northern beans	3.35	168
jalapeno peppers:serrano peppers (1:1)	1.50	75
mango	0.73	36
papaya	0.73	36
zucchini	0.47	24
eggplant	0.47	24
cucumber	0.47	24

Table S1B. Species-level taxa in the Malian diet

Taxa	diet_1	diet_2
Bacteria;Firmicutes;Bacilli;Lactobacillales;Streptococcaceae;Streptococcus;Streptococcus thermophilus	54.08	54.73
Bacteria;Tenericutes;Mollicutes;Acholeplasmatales;Acholeplasmataceae;Candidatus Phytoplasma;Sugarcane phytoplasma uncharacterized	26.78	24.49
Bacteria;Firmicutes;Bacilli;Lactobacillales;Streptococcaceae;Streptococcus;Streptococcus salivarius	6.75	7.40
Bacteria;Firmicutes;Bacilli;Lactobacillales;Lactobacillaceae;Lactobacillus;Lactobacillus casei	3.40	3.45
Bacteria;Firmicutes;Bacilli;Lactobacillales;Lactobacillaceae;Lactobacillus;Lactobacillus paracasei	2.06	2.16
Bacteria;Firmicutes;Bacilli;Lactobacillales;Lactobacillaceae;Lactobacillus;Lactobacillus delbrueckii	1.58	1.65
Bacteria;Firmicutes;Bacilli;Lactobacillales;Streptococcaceae;Lactococcus;Lactococcus lactis	1.45	1.49
Bacteria;Firmicutes;Bacilli;Lactobacillales;Lactobacillaceae;Lactobacillus;Lactobacillus rhamnosus	1.13	1.25
Bacteria;Firmicutes;Bacilli;Lactobacillales;Streptococcaceae;Streptococcus;Streptococcus sp QAUST01	0.88	0.94
Bacteria;Firmicutes;Bacilli;Lactobacillales;Lactobacillaceae;Lactobacillus;Lactobacillus johnsonii	0.37	0.39
Bacteria;Firmicutes;Bacilli;Lactobacillales;Lactobacillaceae;Lactobacillus;Lactobacillus murinus	0.34	0.33
Bacteria;Firmicutes;Bacilli;Lactobacillales;Streptococcaceae;Streptococcus;Streptococcus sp 2010IleoMSXXf	0.29	0.29
Bacteria;Firmicutes;Bacilli;Lactobacillales;Lactobacillaceae;Lactobacillus;Lactobacillus helveticus	0.25	0.27
Bacteria;Firmicutes;Bacilli;Lactobacillales;Lactobacillaceae;Lactobacillus;Lactobacillus animalis	0.16	0.21
Bacteria;Firmicutes;Bacilli;Lactobacillales;Streptococcaceae;Streptococcus;Streptococcus sp 2010IleoMSXIXa	0.00	0.33
Bacteria;Firmicutes;Bacilli;Lactobacillales;Streptococcaceae;Streptococcus;Streptococcus sp 2011OralMSD6	0.00	0.28
Bacteria;Firmicutes;Bacilli;Lactobacillales;Streptococcaceae;Streptococcus;Streptococcus sp 2010IleoMSXVId	0.27	0.00
Bacteria;Firmicutes;Erysipelotrichia;Erysipelotrichales;Erysipelotrichaceae;Ileibacterium;Ileibacterium valens	0.10	0.12
Bacteria;Firmicutes;Bacilli;Lactobacillales;Lactobacillaceae;Lactobacillus;Lactobacillus zeae	0.11	0.12
Bacteria;Firmicutes;Clostridia;Clostridiales;Lachnospiraceae;Blautia;Blautia coccoides	0.00	0.12

Table S1. Composition of the Malian diet

The percentage (w/w) and total amount of each food item used to make the Malian diet (A). The ingredients used were based on observations from the study site and the relative amounts of the ingredients were based on an average from nutritional studies conducted in Mali [1-4]. Abundance of species-level taxa in the Malian diet from two independent samples as a proportion of the total counts (B). Gray shading indicates a value ≥ 1%.

1. Coulibaly A, O'Brien H, Galibois I. Development of a Malian food exchange system based on local foods and dishes for the assessment of nutrient and food intake in type 2 diabetic subjects. South African Journal of Clinical Nutrition. 2009;22:31-5. doi: 10.1080/16070658.2009.11734214.
2. Kennedy G, Fanou-Fogny N, Seghieri C, Arimond M, Koreissi Y, Dossa R, et al. Food groups associated with a composite measure of probability of adequate intake of 11 micronutrients in the diets of women in urban Mali. J Nutr. 2010;140:2070s-8s. doi: 10.3945/jn.110.123612.
3. Parr CL, Barikmo I, Torheim LE, Ouattara F, Kaloga A, Oshaug A. Validation of the second version of a quantitative food-frequency questionnaire for use in Western Mali. Public Health Nutr. 2002;5:769-81. doi: 10.1079/phn2002357.
4. Torheim LE, Ouattara F, Diarra MM, Thiam FD, Barikmo I, Hatløy A, et al. Nutrient adequacy and dietary diversity in rural Mali: association and determinants. European Journal of Clinical Nutrition. 2004;58:594-604. doi: 10.1038/sj.ejcn.1601853.

A

Jaccard distance			
group	time point 1	time point 2	q-value
1DMD	7	14	0.013852
	14	21	0.035298
	21	28	0.173552
4DMD	7	14	0.015607
	14	21	0.118617
	21	28	0.928000
4WMD	7	14	0.016320
	14	21	0.020031
	21	28	0.020031
4W7C	7	14	0.013852
	14	21	0.039152
	21	28	0.185363

B

Bray-Curtis distance			
group	time point 1	time point 2	q-value
1DMD	7	14	0.014191
	14	21	0.052063
	21	28	0.026984
4DMD	7	14	0.022848
	14	21	0.225659
	21	28	0.677000
4WMD	7	14	0.014191
	14	21	0.089282
	21	28	0.132932
4W7C	7	14	0.014191
	14	21	0.103030
	21	28	0.153254

C

unweighted Unifrac distance			
group	time point 1	time point 2	q-value
1DMD	7	14	0.014385
	14	21	0.079508
	21	28	0.250364
4DMD	7	14	0.014385
	14	21	0.396919
	21	28	0.535000
4WMD	7	14	0.014571
	14	21	0.014385
	21	28	0.014385
4W7C	7	14	0.014385
	14	21	0.181679
	21	28	0.373493

D

weighted Unifrac distance			
group	time point 1	time point 2	q-value
1DMD	7	14	0.015915
	14	21	0.051659
	21	28	0.038551
4DMD	7	14	0.020231
	14	21	0.044625
	21	28	0.129865
4WMD	7	14	0.017436
	14	21	0.089282
	21	28	0.034540
4W7C	7	14	0.031552
	14	21	0.381000
	21	28	0.231403

Table S4. Statistics for longitudinal stabilization of the microbiome
The Jaccard distance (A), Bray-Curtis distance (B), unweighted Unifrac distance (C) and the weighted Unifrac distance (D) between subsequent time points in each group was compared by PERMANOVA. Gray shading indicates a q-value < 0.05.

A

Jaccard distance			
group	time point 1	time point 2	adjusted p-value
1DMD	7	14	0.9334
	7	21	0.0095
	7	28	0.0059
4DMD	7	14	0.8264
	7	21	0.9999
	7	28	0.9584
4WMD	7	14	0.9999
	7	21	0.9991
	7	28	0.2673
4W7C	7	14	0.9987
	7	21	0.9634
	7	28	0.6584

B

Bray-Curtis distance			
group	time point 1	time point 2	adjusted p-value
1DMD	7	14	0.9999
	7	21	0.9999
	7	28	0.9996
4DMD	7	14	0.2309
	7	21	0.9999
	7	28	0.9899
4WMD	7	14	0.9999
	7	21	0.9999
	7	28	0.9999
4W7C	7	14	0.2754
	7	21	0.9641
	7	28	0.9999

C

unweighted Unifrac distance			
group	time point 1	time point 2	adjusted p-value
1DMD	7	14	0.9407
	7	21	0.9999
	7	28	0.2386
4DMD	7	14	0.9994
	7	21	0.9999
	7	28	0.9949
4WMD	7	14	0.9999
	7	21	0.9974
	7	28	0.8121
4W7C	7	14	0.9965
	7	21	0.0010
	7	28	0.0119

D

weighted Unifrac distance			
group	time point 1	time point 2	adjusted p-value
1DMD	7	14	0.9999
	7	21	0.9991
	7	28	0.9999
4DMD	7	14	0.9997
	7	21	0.9999
	7	28	0.9124
4WMD	7	14	0.9999
	7	21	0.7750
	7	28	0.8916
4W7C	7	14	0.0870
	7	21	0.9999
	7	28	0.9979

Table S5. Statistics for longitudinal intragroup variation of the microbiome

The intragroup distance for each group at each time point was compared to the intragroup distance at d7 for each respective gavage group, using Jaccard distance (A), Bray-Curtis distance (B), unweighted Unifrac distance (C) and the weighted Unifrac distance (D), by ANOVA with Tukey's multiple comparisons test. Red shading indicates a time point that had significantly increased intragroup distances compared to d7 for that group, and blue shading indicates a time point that has significantly decreased intragroup distances compared to d7 for that group.

A

Jaccard distance			
group	time point 1	time point 2	adjusted p-value
1DMD	7	14	<0.0001
	7	21	<0.0001
	7	28	<0.0001
4DMD	7	14	0.0853
	7	21	0.0023
	7	28	0.0004
4WMD	7	14	0.7318
	7	21	0.7592
	7	28	0.0001
4W7C	7	14	0.9991
	7	21	0.6865
	7	28	0.9999

B

Bray-Curtis distance			
group	time point 1	time point 2	adjusted p-value
1DMD	7	14	<0.0001
	7	21	<0.0001
	7	28	<0.0001
4DMD	7	14	<0.0001
	7	21	<0.0001
	7	28	<0.0001
4WMD	7	14	0.9999
	7	21	0.0340
	7	28	<0.0001
4W7C	7	14	0.0673
	7	21	0.9981
	7	28	0.2209

C

unweighted Unifrac distance			
group	time point 1	time point 2	adjusted p-value
1DMD	7	14	0.5590
	7	21	0.1662
	7	28	0.0004
4DMD	7	14	0.9638
	7	21	0.9515
	7	28	0.7454
4WMD	7	14	0.9944
	7	21	0.5313
	7	28	<0.0001
4W7C	7	14	0.9975
	7	21	0.9999
	7	28	0.9999

D

weighted Unifrac distance			
group	time point 1	time point 2	adjusted p-value
1DMD	7	14	<0.0001
	7	21	0.0027
	7	28	0.0002
4DMD	7	14	0.4867
	7	21	0.9956
	7	28	0.1540
4WMD	7	14	0.9999
	7	21	0.3962
	7	28	<0.0001
4W7C	7	14	0.2882
	7	21	0.3099
	7	28	0.1882

Table S6. Statistics for longitudinal distance to input
The average distance to the input for each group at each time point was compared to the average distance to the input at d7 for each respective gavage group, using Jaccard distance (A), Bray-Curtis distance (B), unweighted Unifrac distance (C) and the weighted Unifrac distance (D), by ANOVA with Tukey’s multiple comparisons test. Red shading indicates a time point where the average distance to input significantly increased relative to the d7 time point for that group.
